# Computer Vision in Monitoring Fruit Browning: Neural Networks vs. Stochastic Modelling

**DOI:** 10.3390/s25082482

**Published:** 2025-04-15

**Authors:** Maria Kondoyanni, Dimitrios Loukatos, Charalampos Templalexis, Diamanto Lentzou, Georgios Xanthopoulos, Konstantinos G. Arvanitis

**Affiliations:** Department of Natural Resources Management and Agricultural Engineering, Agricultural University of Athens, 75 Iera Odos Str., 11855 Athens, Greece; mkondoyanni@aua.gr (M.K.); chartempl@aua.gr (C.T.); dlen@aua.gr (D.L.); xanthopoulos@aua.gr (G.X.); karvan@aua.gr (K.G.A.)

**Keywords:** computer vision, CNN, stochastic modelling, fruit browning, food production chain, agricultural automations, food quality

## Abstract

As human labour is limited and therefore expensive, computer vision has emerged as a solution with encouraging results for monitoring and sorting tasks in the agrifood sector, where conventional methods for inspecting fruit browning that are generally subjective, time-consuming, and costly. Thus, this study investigated the application of computer vision techniques and various RGB cameras in the detection and classification of enzymatic browning in cut pears, comparing convolutional neural networks (CNNs) with stochastic modelling. More specifically, light is shed on the potential of CNN-based approaches for high-throughput and easily adapted applications and the potential of stochastic methods for precise, quantitative analyses. In particular, the developed CNN model was easily trained and achieved an accuracy of 96.6% and an F1-score greater than 0.96 during testing with real pear slices. On the other hand, stochastic modelling provided quantitative indices (i.e., the Browning Index (BI) and Yellowing Index (YI)) derived from the CIE Lab* colour model, thus offering accurate monitoring of enzymatic browning and related optical changes but it was less versatile as it required human expertise for implementation and tuning. Using both the BI and YI as input vectors in the NN Bayesian classifier increased the correct classification rate of control samples to 82.85% (4.6% increase) and to 89.81% (15% increase) for treated samples. Finally, a future need for a hybrid approach combining the strengths of both methods was identified, with improved robustness and practicality of image analysis systems in agricultural quality control to enable higher levels of automation in this area.

## 1. Introduction

The increasing demand for high-quality agricultural products as well as fully automated fruit sorting forced the adoption of advanced technologies for monitoring, sorting, and grading of agricultural products. Human-based methods for processing agricultural products are labour-intensive, subjective, and prone to inconsistencies. In the last decade, developed countries experienced a lack of labour, especially in the agricultural sector. In this context, computer vision (CV) has emerged as a transformative technology, driven by the technological revolution, that can offer automated, objective, and consistent evaluation of agricultural products. Computer vision involves the use of imaging systems, algorithms, and analytical tools to process data extracted from digitally captured images. In agriculture, this technology is employed for tasks such as surface defect detection, ripeness assessment (near-infrared analysis), size grading (visible light analysis), and disease identification (infrared and hyper-/multi-spectral analysis). As the use of most computer vision systems are non-destructive, large quantities of products can be rapidly processed without compromising quality, especially in packing houses where electronic sorting systems are employed [1].

The rapid progress in imaging technologies, covering the perception of hyperspectral, multispectral, and/or thermal information, are facilitating the use of computer vision applications in agriculture. Hyperspectral imaging analysis allows for the detection of subtle biochemical changes in fruits and vegetables, facilitating the early identification of defects and quality deterioration [2]. Additionally, thermal imaging is a very effective method for identifying physiological stress in crops and fruits [3]. The synergy between computer vision, artificial intelligence (AI), and machine learning (ML) techniques has further enhanced the efficiency and accuracy of these applications. Deep learning techniques, like Convolutional Neural Networks (CNNs), have demonstrated good performance in complex image recognition tasks, including the classification of fruit diseases and fruit sorting, based on carefully selected quality characteristics [4]. The abovementioned innovations facilitate the creation of robust tools for addressing the growing challenges in the agricultural supply chain.

Despite these advances, several challenges remain, including the high cost of advanced imaging systems, the need for large datasets for model training, and the variability in environmental conditions, which, along with the difficulty in employing consistent image acquisition protocols in the field, affect image acquisition in an unpredictable way.

One important reason for using a low degree of processing for pear products is that they should retain as much of their fresh qualities as possible, including flavour, texture, and nutrient content. However, this production policy is a significant challenge since pears are prone to enzymatic browning, which negatively impacts their visual appeal, nutritional quality, and marketability. Enzymatic browning is primarily caused by the oxidation of phenolic compounds by the enzyme polyphenol oxidase (PPO) when cellular structures are disrupted during peeling, slicing, or cutting [5,6]. The resulting quinones polymerize into brown pigments. This process is influenced by oxygen exposure, temperature, pH, and the intrinsic phenolic content of the pears. Apart from browning, another challenge is the yellowing of pears, an indictor of quality degradation, which is often associated with chlorophyll decomposition in the pear flesh, revealing carotenoids or other pigments that impart a yellow hue. It should be noted that oxidative stress can accelerate the degradation of green pigments. The factors influencing browning and yellowing include cultivar differences, since some pear varieties (e.g., Bartlett and D’Anjou) have a higher phenolic content, making them more prone to browning; maturity stage, since immature fruits are less susceptible to browning but are more likely to yellow due to their lower phenolic content and higher chlorophyll levels; and storage conditions, since high temperatures and oxygen exposure promote oxidation [7,8,9]. The methods for tackling enzymatic browning include the application of anti-browning agents, from ascorbic acid and citric acid to calcium-based solutions, which inhibit PPO activity or reduce quinones back to colourless compounds. Alternative techniques include using Modified Atmosphere Packaging (MAP) to supress oxidation and reduce oxygen levels, the use of edible coatings, and blanching to inactivate enzymes. It should be noted though, that these approaches must find a good compromise between suppressing browning and maintaining the sensory properties and nutritional quality at acceptable levels. Regardless of the method adopted, identifying enzymatic browning is a major initial step in prolonging the shelf life and commercial success of minimally processed pears and simultaneously responding to the consumer demand for fresh and healthy food products.

## 2. Motivations and Challenges

Conventional methods for evaluating fresh-cut fruit and vegetable flesh browning based on human inspection can be subjective, time-consuming, and costly, especially now when labour is limited and therefore expensive. Many techniques have been proposed and used to evaluate fruit browning, including the use of colourimeters, spectrophotometers, image analysis, which generate large datasets that should be processed in a short amount of time due to processing time limitations. But even if these techniques use special algorithms that can be executed by dedicated (embedded) computers installed in the premises, challenges still exist. In fact, a significant disadvantage of this methodology is that the accurate performance of the final system depends heavily on the contribution of food and signal-processing experts and the adaptation to different fruit product line requirements requires a considerable number of changes to the pre-existing algorithm. Adapting these systems to various food products often necessitates substantial changes to the existing algorithms, posing practical challenges in diverse processing environments [10].

On the other hand, machine learning (ML), as an alternative methodology for image data processing, presents a viable solution to address these challenges. ML techniques provide real-time applicability within processing lines and accommodates the analysis of considerable product volumes. Machine Learning Algorithms (MLAs) can automate repetitive tasks and processes, leading to improved efficiency and reduced human effort. This is particularly useful in handling large datasets and complex tasks. Furthermore, MLAs can extract valuable information and patterns from large datasets that are difficult for humans to process manually. MLAs can process visual information in real time, making their operation suitable for applications that demand fast decisions. Moreover, the scalability of a trained ML model can be adjusted in terms of accuracy and hosting computer resources, allowing its adaptability to a wide variety of contexts. The rapid development of the electronics industry has facilitated an increase in the quantity and quality of several components, including Microcontroller Units (MCUs), single-board computers, sensors, and radio transceivers, which have rendered these intelligent methodologies economically viable. Specifically, the latest generation of microcontrollers, in addition to performing typical sensing and actuation tasks, can support complex operations with reduced execution times due to their faster and more efficient processors and greater memory.

These advancements favour the utilization of machine learning, especially through the use of CNNs, in a large variety of applications and projects. CNNs have emerged as a powerful tool in image processing, achieving high performance in a variety of areas from visual data. Since this advancement, CNNs have transformed the way computers interpret visual information [11]. They were originally developed for features extraction for tasks such as image classification or segmentation; CNNs have been widely used in all areas of computer vision where high accuracy is required. Their success can be attributed to their multilayer structure, where convolutional layers capture spatial features from the images. For tasks using RGB images, simpler CNN architectures are usually used due to their ability to extract visual features based on colour [11,12,13].

Moreover, the uniform and generic manner in which these techniques can be applied have enabled their implementation and deployment by personnel without significant expertise in the field. The scalability of deployment translates into MLAs operating on endpoints that host a specific CV system. These devices can be low cost and small in size and support energy-saving schemes and/or harvesting options, extending their operation lifespan. Their processing capability and memory capacity have been enhanced so as to support MLAs at a negligible additional financial cost. Furthermore, the local execution of ML algorithms results in significantly reduced energy and networking requirements compared to the conventional centralized system approach.

Another factor that enhances the feasibility of utilizing CNNs on microcontroller units (MCUs) is the advancement in the corresponding tools for training, programming, and deployment, along with software platforms that provide high accessibility for users. It is noteworthy that the training phase of CNNs is considerably more computationally demanding than the execution phase. Once the CNN has been trained, the corresponding model being deployed can be run on computers with many times less computational power and memory than the machine used for the training. This is consistent with the constraints of MCUs with very limited resources. The AI models can be generated through a fine-tuning process that takes advantage of pre-trained CNNs and the use of additional sampled data to improve the overall performance without having to rebuild everything from ground zero. It should be noted that certain trained models may still present a potential overhead for MCUs. However, there is the option to produce streamlined versions of these complex models that are compatible with the newer generation of MCUs [14]. This task can be accomplished with the assistance of user-friendly tools such as TensorFlow Lite [15] within the context of the TinyML paradigm [16].

This approach involving AI enables computations to be performed in proximity to the data collection point and was employed in the experiments in this study. The concept of in situ processing and decision making, as expressed by the Edge Computing (EC) term, and its apparent advantages serve to complement the performance of the abovementioned tasks with additional benefits. Specifically, unlike traditional cloud computing, which requires data to be uploaded to the cloud, EC mitigates risks such as data loss while simultaneously enhancing security and privacy since the critical data are used locally and remain within the premises of the firm. Furthermore, edge computing results in reduced energy and network bandwidth consumption [17]. The deployment of artificial intelligence in an edge computing environment is referred to as Edge AI [18].

To offer a solution to the abovementioned challenge of improving performance, this study focused on the identification of browning via computer vision and its limitations by comparing and evaluating two different methodologies. The first uses neural networks for complex image recognition tasks, such as fruit sorting. The second one is a more conventional counterpart that includes stochastic modelling, which was based on theoretical approaches and physical principles, and uses colour models for the assessment of quality attributes and other visual problems.

## 3. Materials and Methods

In line with the dynamics described above, this section presents in detail the two methodologies used (the CNN-based and the stochastic one) to classify fresh from enzymatically browned pear slices. The data prepared for the experiments were used with both methodologies. The collection process, as well as the sensors used for the dataset creation, is described in Section 3.1. Section 3.2 provides information about the AI-based approach, which includes an outline of the steps in the development of the ML model, a description of the data pre-processing techniques used prior to the training, details on the platform used for the training and the basic programming, along with the components used for the on-device implementation (i.e., sensors and microcomputer) that facilitated the real-time data analysis. An analysis of the stochastic approach is given in Section 3.3. Specifically, the pre-processing of the data is described, which includes techniques for image thresholding and segmentation, the CIE Lab* model is presented along with the Browning and Yellowing indices, and finally, information is provided about the statistical analyses that were conducted.

### 3.1. Data Preparation

**Figure 1:**
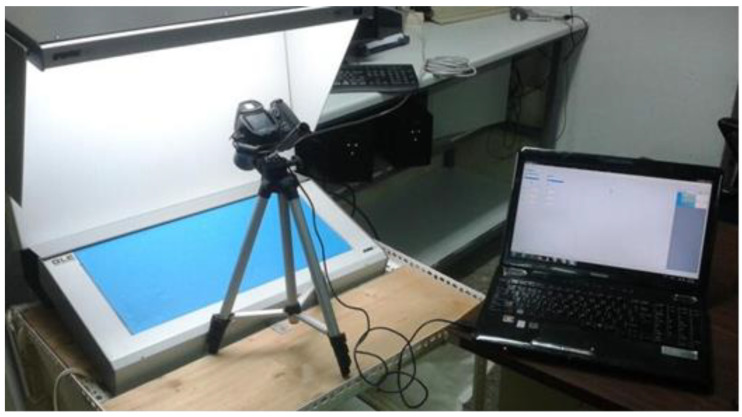
Digital image acquisition system and image analysis software.

The digital images were captured in a light booth utilizing the appliance Graphic Lite GLE PDV-3eTR (made by Graphic Technology, Inc., New York, NY, USA), equipped with a D65 lighting system (L317, Graphic Technology, Inc., New York, NY, USA) (Figure 1). The D65 fluorescent lamps were selected according to ISO/CIE 11664-2:2022 (CIE standard illuminants for colourimetry) [19] to simulate daylight. A Konica Minolta DiMAGE Z6 (Konica Minolta, Inc., Tokyo, Japan) [20] digital camera was employed with the default settings: exposure time (shutter speed) of 1/15 s, lens brightness of f.5, and the highest level of image quality (high quality) with dimensions of 640 × 480 pixels [21]. The horizontal and vertical resolutions were 72 dpi, and shooting was performed with zero focus without the use of a flash [22]. The colour representation of the digital images was based on the RGB colour model. The shooting angle was the 10° Standard Observer according to ISO/CIE 11664-1:2019 (CIE 1964 10° standard colourimetric observers for colourimetry) [23]. For agricultural applications, the CIE 1964 10° Standard Observer [24] is generally preferred to the CIE 1931 2° Standard Observer [24] because it provides a more representative colour perception for large, natural surfaces such as fruits, leaves, and crops. Many agricultural imaging systems and colourimeters use it to obtain better accuracy in assessing plant health, fruit ripeness, and food quality. Since agricultural samples are typically larger than the 2° foveal vision field, the 10° Observer is more appropriate. In cases where small details are being analysed (e.g., microscope imaging of plant tissues), the CIE 1931 2° Observer [24] is preferred.

**Figure 2:**
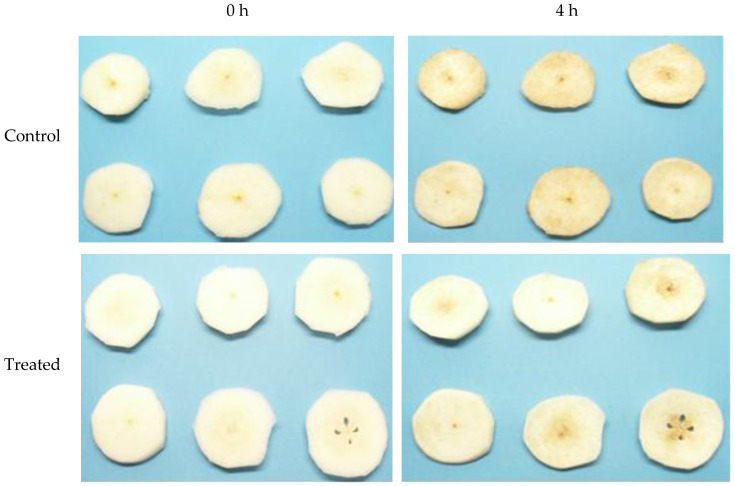
Control pear slices vs. those treated with an aqueous solution of ascorbic and citric acid at 0 (**left**) and 4 h (**right**) after the start of the experiment.

The samples were photographed at time 0 (when they were freshly cut) and then every 30 min for 9 h (18 measurements). Two representative images of each class (control pear slices vs. those treated with antioxidants) from the digital image dataset are presented in Figure 2. As evidenced by the images in Figure 2, the slices treated with the antioxidant solution exhibited preservation of the pear slices’ visual integrity compared to the control sample after a four-hour exposure to air. The objective of this study was to employ image analysis techniques to quantitatively and objectively determine these discrepancies in visual quality. In this study, image acquisition was carried out under almost constant lighting conditions and with specific equipment to maximize the image acquisition accuracy and minimize environmental interference. This setup matches the specifications utilized by the food industry in food production lines where sorting procedures take place. Considerably different lighting intensities, colour temperatures, and on-site background interference were not considered as they are also present in real-world applications. The specific and strict protocols for image acquisition and processing in terms of lighting, observer angle, and imaging background are used by commercial electronic vision produce graders [25].

### 3.2. AI-Based Approach

#### 3.2.1. Machine Learning Steps

**Figure 3:**
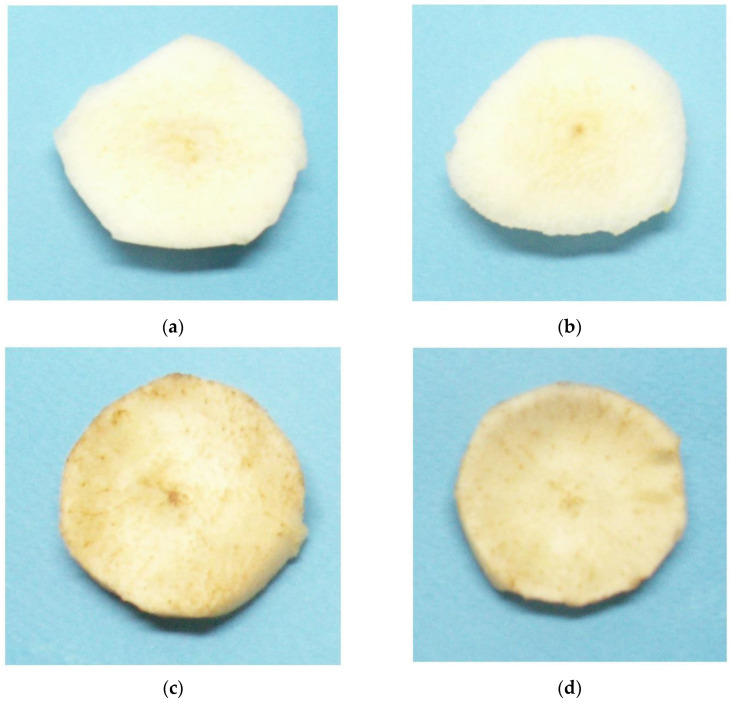
Images of pear slices from the two labelled classes used for supervised learning: (**a**,**b**) were classified as “good”, while (**c**,**d**) were classified as “brown”.

In this section, the methodology designed to discern distinct classes of pears using a neural network is outlined. The primary objective of this method is to distinguish between fresh and browned pears due to oxidative browning. This is an unfavourable outcome for customers, particularly when the pears are used in fresh fruit salads. For this reason, a machine learning model was developed with the objective of classifying the pear slices and indicating the optimal utilization of them, with the aim of preventing wastage during post-harvest processing in food manufacturing. In the AI-based approach, the principal objective was to develop a neural network (NN) model to distinguish freshly cut pears with white flesh from those with brown spots. The methodology consisted of a number of stages. In order to generate a dataset, initially, digital images from pear slices were taken and then labelled (Figure 3). The segmentation method in the following step was optimized for the digital images in order to facilitate the feature extraction phase. Then, a pre-trained NN was employed to achieve the optimal feature extraction. In the final stage, a ML classifier was trained using the deep features extracted by the neural network to classify pears into two categories designated as “good” and “brown”. The dataset employed comprised a diverse array of images featuring a variety of pear slices. Finally, the trained NN model was deployed on a Raspberry Pi for real-time classification of pear slices.

In this phase of the experiment, only untreated slices of pears were used since the objective was to adopt a more comprehensive approach to the real-time classification of fresh and non-fresh pear slices. The use of pear slices treated with an aqueous solution of ascorbic and citric acid was avoided as they would have introduced additional variables into the experiment. The images were subsequently employed for the development of the ML model. The development of the ML model was facilitated by the user-friendly cloud platform Edge Impulse [26]. After properly adjusting the parameters for the training process, the model exhibiting the best performance was selected for deployment into the dedicated hardware. The principal component for deployment was a Raspberry Pi 4 microcomputer [27], which served as the central processing unit and facilitated experimentation with three different digital cameras. In the primary scenario, the Raspberry Pi, in conjunction with a basic USB webcam [28], was employed. As an alternative, an OV5693 USB camera (OmniVision Technologies, Inc., Santa Clara, CA, USA) with 5MP resolution [29] was utilized to investigate whether the system’s accuracy had been compromised. Finally, the last test was conducted without the Raspberry Pi, employing a smartphone [30].

#### 3.2.2. Training Details and On-Device Model Integration

The data that were meticulously gathered according to the image capturing method described in Section 3.1 resulted in 108 figures containing one pear slice each, which ranged from white to considerably browned ones. These figures were manually divided into three categories, with almost equal numbers of photos in each category. The first category contained pear slice photos that were taken during the first third of the image capturing process and thus, these slices were in good condition, i.e., more white in terms of colour. The second category contained photos of pear slices in medium condition. The third category contained pear slice photos that were taken during the last third of the experiment. From these categories, the second one was left out, while the other two were utilized to form the two main classes of the neural network model. More specifically, prior to initiating the training process, the photos corresponding to each of these two categories were divided into two subsets using a random pick algorithm in the Edge Impulse platform. The Edge Impulse platform recommends utilizing 20% of the dataset as the “testing” dataset, although this percentage may vary slightly based on whether the total amount of collected samples is divisible in this way. Therefore, the division was executed across a range of 78–22%, without compromising the model’s performance or efficiency. The first subset was used for the main training stage, while the other was used for the testing, i.e., the initial evaluation of the model’s efficacy. Once the requisite data had been collected and divided into the subsets, the next step was to design and train the model. This entailed the addition of a “processing block” that modifies the data and a “learning block” that enables the selection of the specific NN to be trained. As the dataset consisted of RGB images, the “image” block was selected as the optimal choice for the “processing block” due to its ability to pre-process and normalize image data, as well as to reduce colour depth. Additionally, the pre-processing steps, i.e., before the main training process, included image cropping and/or resizing to ensure that all the images were equally sized and aligned with the shortest axis. More specifically, an image size of 160 × 160 pixels was selected to ensure optimal compatibility with the transfer learning requirements and to achieve a good balance between satisfactory accuracy and a reasonable size and degree of complexity for the trained model. Regarding the “learning block”, transfer learning was applied, which is a pre-trained image classification model that can work well even with relatively small image datasets, such as the one utilized in the research being presented.

The “processing block” of the Edge Impulse engine [26] generated 76,800 features, which were used to form the input layer of the NN structure. The number of training cycles was set to 20, the learning rate was set at 0.0005, and the output layer contained two neurons corresponding to the brown and the acceptable pear slices. The model comprised two classes as its use is intended solely for the sorting of pears slices in production lines and the decision is made at the next stage when the set similarity threshold is exceeded. During the training process, a sequence of data augmentation techniques was systematically implemented using the Edge Impulse platform to mitigate the risk of overfitting and improve the model’s accuracy. These techniques entailed the random transformation of data instances throughout the training process. The model employed was MobileNetV2 [31], a deep neural network architecture designed for efficient and lightweight classification of images, reducing computational and memory usage, and thus making it an ideal choice for real-time applications on resource-constrained devices [32], like mobile and embedded devices. The MobileNet was selected over other CNN architectures as it is suitable for lightweight models implemented on edge devices, providing fast inference and good accuracy. On the other hand, other variants work better for different applications, such as U-Net, which is designed for image segmentation, and ResNet as well as EfficientNet, which are suitable for deep models with high accuracy but they run slower than MobileNet. The specific MobileNetV2 architecture used for the training requires input images with dimensions of 160 × 160 pixels. This configuration translated to a memory usage of approximately 683.3 kilobytes (KB) of random-access memory (RAM) and 658.4 KB of read-only memory (ROM) when using standard settings. With this model, the optimal performance was achieved for input data comprised of RGB images with dimensions of 160 × 160 pixels. Following the training process, the Edge Impulse software [26] saves the model exhibiting the best performance in the Quantized (int8) version, which is compatible with the Raspberry Pi hardware platform (Raspberry Pi Foundation, Cambridge, UK) [27]. Edge Impulse retrieves the model in either a code-based (library) format or as an executable entity. The latter approach was selected, enabling the model to be executed in real-time scenarios.

**Figure 4:**
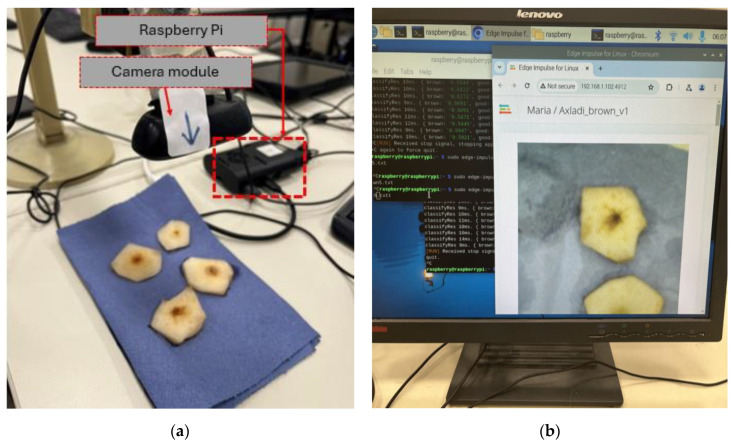
On-device model integration: (**a**) overview of the experimental set-up; (**b**) software arrangements during testing.

A key step in the development of the NN model is its integration into the suitable end device. In the main scenario, a Raspberry Pi 4 single-board microcomputer was utilized to execute the model, while a standard USB camera was employed to provide the visual input in real time. The necessary dependencies were installed on the Raspberry Pi in accordance with the instructions provided by Edge Impulse. To ensure compatibility with the USB camera hardware, the 32-bit “Raspberry Pi OS” was installed. Specifically, a binary add-on for Linux that implements the Edge Impulse Linux protocol, Linux ARMv7, was downloaded from Edge Impulse. Apart from this, in conjunction with the Edge Impulse command-line interface (CLI), the “Εdge Ιmpulse Μodel” (EIM) file (containing the entire trained model) was downloaded and invoked for execution. It should be noted that, for facilitating the execution of the model by non-expert users, the Edge Impulse provides a cloud-based alternative for the execution of the model. Once the model was installed on the Raspberry Pi, two windows were deployed, one displaying the camera’s footage and one showing the prediction outputs and the probabilities assigned by the model to each class (Figure 4).

In addition to the Raspberry Pi (Raspberry Pi Foundation, Cambridge, UK) [27] and the basic Logitech HD C270 webcam (Logitech International S.A., Lausanne, Switzerland) [28], another USB camera [29] was also evaluated using the same configuration and a test with an iPhone 13 smartphone [30] was conducted to assess the efficacy of the ML model on a range of devices with different processors and digital cameras. In the case of the smartphone scenario, the model was deployed in the cloud. This feature of the Edge Impulse platform facilitates experimentation but has limitations, as it is inherently dependent on cloud computing and requires an internet connection. For this reason, in this study, effort was made to address these challenges at the edge, thus eliminating dependencies on cloud resources.

### 3.3. Stochastic Approach

#### 3.3.1. Image Thresholding

Although the imaging background was selected based on the chromatic contrast with the surface colour of the pears, shading at the boundary of the samples was visible, causing inaccuracies during the image analysis. Consequently, in the image processing step, the edge shading around the pear slice was removed by employing Corel PaintShop Pro 2023 [33] and the imaging background was substituted digitally with magenta, which has L* = 61, a* = 98, and b* = −61 values that were used as the threshold values. The digital images, in which six pear slices were included, were split into six images, each containing a single slice. This process yielded a total of 228 digital images.

#### 3.3.2. Image Segmentation

**Figure 5:**
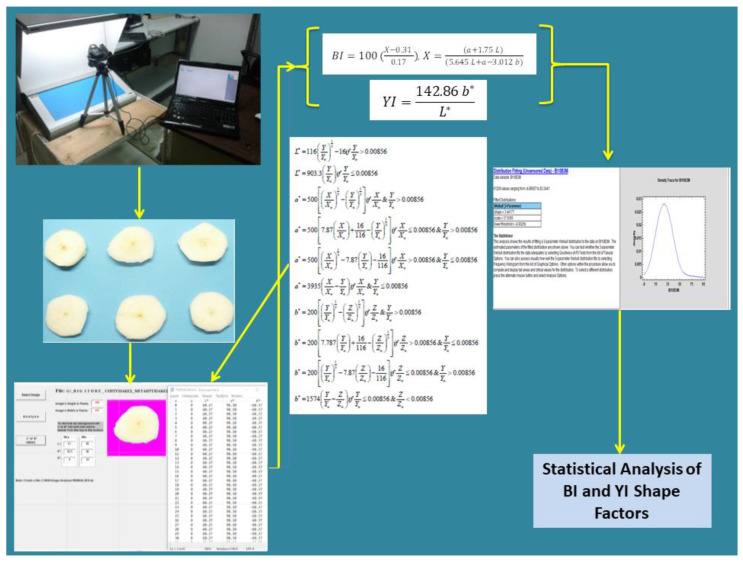
Flowchart of digital imaging analysis.

Image segmentation is the process of grouping similar regions or parts of a digital image. A digital image is regarded as being composed of one or more sets of pixels, which are classified into one or more categories. Consequently, the task of structuring analogous parts of an image is equivalent to pixel-level classification. Image segmentation is an improvement of the digital image categorization and is used for both the classification of images and the precise identification of the location and boundaries of objects. Using this method, the 228 images were processed using the magenta threshold values (i.e., L: 60, 61; a: 98, 98.8; b: −62, 0.0). The segmentation was facilitated by employing the Image Analysis software developed by the Farm Machinery Laboratory of the Agricultural University of Athens (Patent Number: 100655) [22]. The derived L*a*b* data were then employed in the calculation of the BI and YI values and the subsequent mathematical analysis (see Figure 5).

#### 3.3.3. CIE L*a*b* Model

The CIE L*a*b* colour model, also known as CIELab, was first introduced by the International Commission on Illumination (CIE) in 1976. This model is derived from the CIE XYZ colour space and is considered to be a visually uniform colour space that best matches all colour systems or models of human perception of colour differences. Each colour is described by three indices (tristimulus colour model or RGB colour model) and is influenced by the Munsell system [34]. The method for converting the R, G, and B (sRGB) values of the pixels, recovered from the digital photos, to the values of the chromatic parameters L*, a*, and b* according to the values of the variables defined by the standard D65 lighting system and the 10o shooting angle, as well as the standard equations used in the analysis software code, was described by Chatzis et al. [22]. The RGB colour space cannot be directly transferred to the L*a*b* colour space. Two calculation steps are needed to implement the conversion. The RGB colour space is initially transformed into a specific CIE XYZ colour space. The L*a*b* colour space then is defined relative to the tristimulus values of the reference white point (X_n_, Y_n_, Z_n_) of the XYZ space from which they were converted.

Colour analysis is a fundamental tool in food science, particularly for monitoring browning reactions in fruits caused by enzymatic and non-enzymatic processes. The following are the most commonly used colour spaces for food analysis:

- CIE Lab* is the most widely used colour space to monitor food and fruit browning because is objective, uniform, device-independent, and reproducible across different imaging systems and matches human vision, allowing for the quantitative tracking of colour changes due to browning. It is used in image analysis and the analysis of spectrophotometry and colourimeter data. Applications of this model have showed that browning is negatively correlated with L* (as fruit browns, it gets darker), and a* shifts toward red and b* increases as browning progresses.
- CIE LCh° is better for hue and chroma interpretation. It is derived from the Lab* colour model but is more intuitive for colour perception as it uses chroma (C*) and hue angle (h°) instead of a* and b*. Browning results based on this model have showed that as browning progresses, h° shifts toward yellow-red indicating oxidation, and C* decreases as brown pigments reduce the colour vibrancy.
- CIE XYZ is a fundamental colour model for spectrophotometry. This model is the basis for all CIE colour models and is used in spectrophotometers and hyperspectral imaging. For fruit browning analysis, the raw reflectance data of browned fruits are transformed into XYZ values. This colour model is useful for advanced colourimetry and light interaction studies but it is not suitable for visual interpretation.
- RGB and HSV are used for image processing in digital analyses and are used in computer vision, hyperspectral imaging, and digital food analysis (ripeness, colour-based sorting, identifying defects). Although they are simple, they are very sensitive to lighting conditions (device-dependent since values change with different cameras, lighting, and sensors). Using the RGB model, fruit browning is reflected as a reduction in R/G values and an increase in B (due to blue-darkening effects). In the HSV colour model, fruit browning often shifts the hue from yellow/orange (fresh fruit) to brown, the saturation decreases due to oxidation and enzymatic reactions, and browning areas tend to appear darker due to pigment changes affecting the value (V) which represents the brightness.
- Hunter Lab is similar to CIE Lab* but is less standardized and stands as its alternative model with slightly different calculations. This model is used in food processing, quality control, and industrial applications [35,36].

Thus, it was concluded that for the fruit browning analysis, CIE Lab* was the best standard because (i) it accurately tracks browning trends (decrease in L*, shift in a*, and increase in b* with increased browning), (ii) it is widely accepted in scientific and industrial food studies and, (iii) it can be used for both spectrophotometry data and image processing.

#### 3.3.4. Browning and Yellowness Indices

The Browning Index (BI) is a widely used metric that describes the changes that occur during the browning process of horticultural products. The index is defined as the purity of the brown colour and is considered to be one of the most reliable indicators of browning in edible agricultural products, especially in fresh-cut produce. The BI method quantifies the intensity of browning and focuses on the red-brown hue associated with enzymatic and non-enzymatic browning. The metric is primarily influenced by changes in the a* (+a = red, −a = green) and b* (+b = yellow, −b = blue) components of the CIE Lab* colour space, as well as the lightness L* (0 = black, 100 = white). The X variable in the BI equation (Equation (1)) is a chromatic parameter that represents a weighted combination of the L, a, and b values. The X variable is dimensionless and reflects the relative contributions of lightness (L), red–green (a), and yellow–blue (b) in browning processes. The denominator normalizes the equation to avoid extreme values under different colour conditions. Pathare et al. [34] presented the index as follows:

(1) $$BI=100(\frac{X-0.31\;}{0.17}),\;X=\frac{(\alpha^{*}+1.75\;L)\;}{\left(5.645\;L+a-3.012\;b\right)}$$

The Yellowing Index has been associated with scalding, contamination, and general deterioration of agricultural products due to the effects of light, chemical exposure, and processing. The Yellowing Index is predominantly employed to quantify these types of degradation with a single value on an arbitrary scale. The Yellowing Index (YI) has been described as a measure of the degree of yellow colouration [37] and is calculated as follows:

(2) $$YI=\frac{{142.86\;b}^{*}}{L^{*}}$$

Both the BI and YI indices are dimensionless. The BI remains a standard and reliable metric for assessing fruit browning, especially enzymatic browning, while alternative methods like image analysis and high-throughput colour measurement systems also offer promising, efficient, and non-destructive means of evaluation. The choice of method should consider factors such as the specific type of browning, the fruit variety, the available equipment, and the required precision for the assessment. This highlights the need for further work in this field, but this topic is outside the scope of this study.

#### 3.3.5. Statistical Analysis and Weibull Tri-Parametric Distribution

The statistical analysis of all the data from the image analysis was conducted using the Statgraphics 19 software (Statgraphics Technologies Inc., The Plains, VA, USA). The Browning and Yellowing indices for each digital image were calculated using the Statgraphics 19 statistical software. The shape factor for each case was calculated using the Weibull three-parameter distribution. The goodness-of-fit tests for the BI and YI values indicated that the lowest *p*-value among the tests was less than 0.05, thereby supporting the hypothesis that the BI and YI values can be derived from the Weibull tri-parameter distribution with a 95% confidence level.

This distribution is a continuous probability distribution function that describes the behaviour of systems exhibiting time variability. The probability density function of a Weibull random variable is described mathematically below.

(3) $$\begin{array}{cc} f\left(x\right)=\left\{\begin{array}{c} \beta \\ \alpha \end{array}\right.+{\left(\frac{x-\gamma }{\alpha }\right)}^{\beta -1}\;exp\left[-{\left(\frac{x-\gamma }{\alpha }\right)}^{\beta }\right], & x>0\\ 0, & x\;\leq 0 \end{array}$$

where α > 0 and is the scale factor (or characteristic life), defined as the reaction rate constant; β > 0 and is the shape factor (or slope), defined as the behavioural index; γ the location parameter (or failure-free life). By varying the value of γ, the distribution slides either to the right (γ > 0) or to the left (γ < 0). The distribution displays first-order decay kinetics when β = 1, and the failure rate increases as a function of time if β > 1 and decreases as a function of time if β < 1. 

The dimensionless shape parameter (β) determines the shape of the curve, so for β < 1, the failure rate decreases with time; if β > 1, the failure rate increases as a function of time, suggesting that an “ageing process” is in progress. High β values indicate that failure will take place within a relatively short time period, while if β = 1, the failure rate is constant [38]. The Weibull distribution has been efficiently used in agricultural applications such as seed germination failure due to genetic defects (β < 1), degradation of fruits and grains during storage, and mechanical fatigue of agricultural equipment (β > 1). In postharvest studies, it has been used to efficiently determine the spoilage rates of fruits, vegetables, and grains [38,39]. Thus, the implementation of this methodology provides not only a grading/selection operation but also quantitative data that are useful for decision-making processes.

The linear models for BI and YI = ƒ(exposure time) were assessed according to four statistical hypotheses concerning the calculated residuals [e_i_ = (YI, BI)_exp_ − (YI, BI)_pred_] of the regressed model.

i. E(e_i_) = 0 is an ideal model that generates zero residuals; therefore, the smaller the residuals, the more efficient the model. The hypothesis was tested using error expressions.

ii. var(e_i_) = σ^2^ is the homoscedasticity hypothesis where the variance σ^2^ of the residuals is constant over the entire range of the residuals. The hypothesis was tested visually by plotting the residuals against the predicted values. The residuals must exhibit a non-systematic pattern according to the tested model.

iii. cov(e_i_, e_j_) = 0 for every i ≠ j (adjacent measurements) is the autocorrelation hypothesis, which was tested visually by analysing the autocorrelation plot.

iv. ē ≈ N_n_(0, σ^2^I_n_) was used to inspect the normality of the residuals.

## 4. Results

In this section, the results of the experiments performed with the two methodologies are presented. In Section 4.1, the performance evaluation results of the CNN model using on-device implementation and the relevant metrics are presented, while in Section 4.2, the statistical analysis of the BI and YI is shown. The experiments were designed to show the capabilities of our approaches to support our key claims, which were that (i) both methodologies are suitable for browning detection tasks, (ii) we can design an embedded system for common classification tasks in the agri-food industry utilizing cost-effective sensing modules, (iii) the accessibility and affordability of small but powerful hardware components and the availability of lightweight neural network models can facilitate the development of systems that perform real-time classifications, and (iv) the stochastic methodology not only provides qualitative results, but also the rate of browning in quantitative terms as well.

### 4.1. Neural Network Model Performance

In the context of MLA evaluation, accuracy is the most commonly employed indicator. It quantifies the proportion of accurate results, both true positives (TP) and true negatives (TN), among the total number of cases examined; it is expressed as

(4) $$accuracy=\frac{TP+TN}{TP+FP+FN+TN}$$

**Figure 6:**
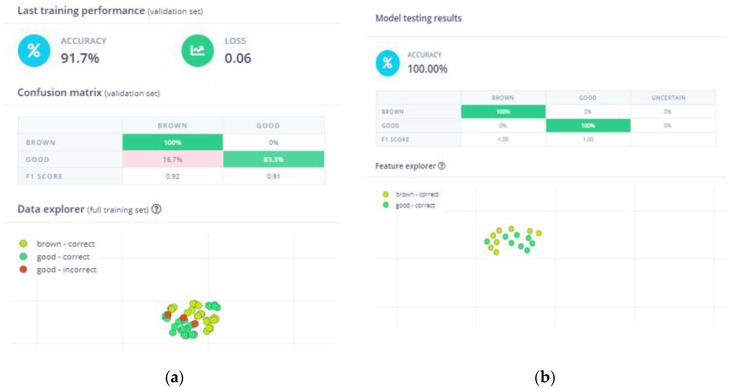
ΝΝ model performance: (**a**) accuracy and confusion matrix of the model generated by Edge Impulse after training; (**b**) accuracy and confusion matrix generated by Edge Impulse based on the images in the testing dataset.

In our case, the model exhibited an accuracy of 91.7% following training (see Figure 6), which was considered sufficient for an ML model with a limited dataset. The preliminary testing, conducted with images that were excluded from the dataset for this purpose, yielded an accuracy of 100%. Accordingly, these metrics were deemed sufficient to justify proceeding with the deployment. For the implementation stage, apart from the necessary electronic equipment, real pear slices were prepared in order to experiment with the ML model and evaluate its performance under real-world conditions. It is worth noting that the experiment set-up was different from the one used for the data acquisition (i.e., varying the lighting conditions and capturing angles), which increased the level of difficulty.

**Figure 7:**
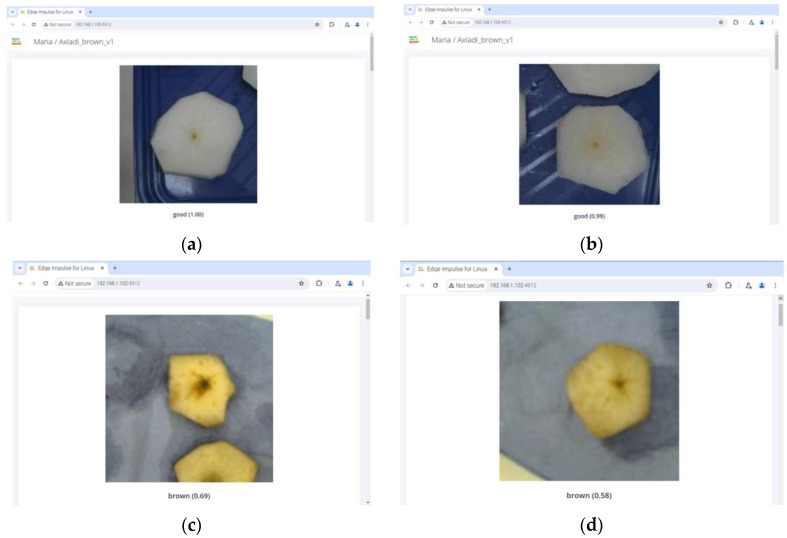
Screenshots of the image classification using the deployed model, the Raspberry Pi, and the Logitech camera. The top two images (**a**,**b**) present pears classified as “good” according to the NN model, while the bottom two images (**c**,**d**) show pears classified as “brown”.

In the primary scenario, the Logitech HD C270 (Logitech International S.A., Lausanne, Switzerland) [28] was utilized to capture images of several pear samples of different varieties, which were subsequently processed in a manner consistent with the image collection phase. Figure 7 shows the cut slices during the testing phase.

Using this configuration for both fresh and brown pear samples, a total of 4080 predictions were made. These real-time predictions generated by the NN model were stored in a text file to facilitate the subsequent analysis. The accuracy of the testing for the 4080 cases was 96.6%, which was quite encouraging as it surpassed the theoretical evaluation. However, to obtain a more comprehensive evaluation, additional metrics were calculated, including sensitivity, precision, and the F-measure, in addition to accuracy. A confusion matrix was constructed to illustrate the outcomes of true positives (TPs), false positives (FPs), false negatives (FNs), and true negatives (TNs) [40]. Table 1 shows the confusion matrix for the binary classification, which was used as the basis for the results table in this experiment.

As can be observed from Table 2, the most prevalent error during the evaluation of the deployed model was the misclassification of browned flesh as good. This misclassification is understandable given that images in this category exhibits characteristics associated with fresh-cut pears, albeit with some brown spots. On the other hand, no erroneous predictions were made regarding fresh slices, which was a highly encouraging outcome, since such errors would suggest potential deficiencies in the model’s design.

As previously discussed, in order to perform an accurate evaluation of a model, it is advised that additional metrics should be calculated [41,42]. Therefore, based on the aforementioned results, the following three metrics were calculated and are presented in Table 3 with the corresponding formulas provided for reference:

- *Sensitivity (or recall)* measures how effectively a classifier can recognize the positive samples. The sensitivity was calculated as follows:
  (5) $$sensitivity\;(or\;recall)=\frac{TP}{TP+FN}$$
- *Precision* is the ratio of correctly predicted positive examples to the total number of predicted positive examples in a given class and was calculated as follows:
  (6) $$precision=\frac{TP}{TP+FP}$$
- The *F1-score* is the harmonic mean of the sensitivity and precision:
  (7) $$F1-score=2\times \frac{precision\times recall}{precision+recall}$$

The model exhibited high precision for both the “brown” and “good” classes, indicating a low incidence of false positive errors and a high degree of accuracy in its classification. Furthermore, the model demonstrated perfect identification/recall for the “good” class, indicating that it successfully identified all the “fresh” samples. Similarly, the recall for the “brown” class was high, indicating that the model correctly captured most of the “brown” samples, although a small proportion was still incorrectly classified. This may be because the browned pears have characteristics similar to fresh pears in terms of colour and texture, whereas the opposite is not the case. Finally, the F1-scores for both classes were similar and high, reflecting a balanced performance between precision and recall. This balance implies that the model functions effectively, minimizing the occurrence of false positive and false negative errors, while accurately identifying true positive cases.

### 4.2. Statistical Analysis of BI and YI

**Figure 8:**
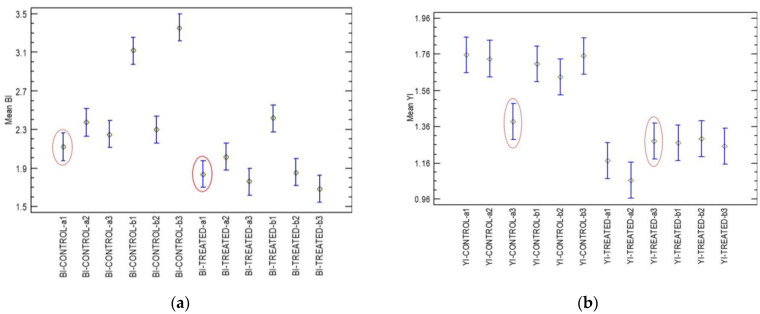
ANOVA of BI (**a**) and YI (**b**) shape factors for control and treated pear samples. Mean values are from Fisher’s LSD test (*p* ≤ 0.05). Sample pairs (control vs. treated) exhibiting no significant differences are circled in red.

The shape factors of BI and YI were statistically analysed using Fisher’s Least Significant Difference test (LSD) and Statgraphics 19 [43] to determine if there are any significant differences (*p* ≤ 0.05) among the mean values. For the BI and YI shape factors, one pair was identified from the test samples (control vs. treated) that was not statistically significant, although different pairs were identified for the two shape factors. Thus, for the BI shape factor, the “a1” pair of samples was not significantly different, while for the YI, the a3 pair was not significantly different (Figure 8a,b). Therefore, employing the shape factors of both indices efficiently removed the uncertainty in the identification of the real differences between any pair of samples.

Both sets of BI and YI were also analysed individually using the NN Bayesian classifier of Statgraphics 19. The NN was trained employing the *Jackknife* cross-validation method, which is a form of re-sampling that is useful for bias and variance estimation. With regard to the BI shape factor, 71.29% of the control samples (108 in total) were correctly classified, while 79.63% of the treated samples (108 in total) were correctly classified. In the case of the YI shape factor, 85.18% of the control samples (108 in total) were correctly classified, while 70.37% of the treated samples (108 in total) were correctly classified. The implementation of the shape factors for the YI and BI in the image analysis normalized the L*, a*, and b* data obtained from the pear digital images, minimized the data variability, and described their temporal variation using a simple mathematical model [YI, BI = ƒ(t)]. When both the BI and YI were used as input vectors in the NN Bayesian classifier, 82.85% of the control samples (108 in total) were correctly classified, while 89.81% of the treated samples (108 in total) were correctly classified. Employing both the BI and YI as input vectors, increased the classification accuracy by an average of 4.6% for the control samples and 15% for the treated samples. The increase in the classification accuracy when the BI and YI were both used as input vectors, rather than one at a time, in the Bayesian classifier for pear browning assessment was attributed to a series of key factors: (i) Complementary information. The BI and YI describe different aspects of pear browning, which may be related to different physiological and biochemical processes. The browning process has been associated with enzymatic oxidation (e.g., polyphenol oxidase activity) or physiological disorders, whereas yellowing has been associated with ripening-related pigment changes (e.g., chlorophyll degradation or carotenoid synthesis). By combining the BI and YI, the Bayesian classifier can better distinguish between different causes and stages of pear browning. (ii) Multivariate relationships. The association between the BI and YI can be complex and dependent on factors such as the storage conditions, cultivar characteristics, or postharvest treatments. A Bayesian classifier uses the joint probability distribution of the BI and YI, allowing it to identify subtle interactions that are missed when the two indices are treated separately. (iii) Improved feature space representation. Considering the BI and YI together extends the feature boundaries, improving the classifier’s ability to distinguish between different browning severities and causes. This higher-dimensional representation allows for better class separation and increasing classification accuracy. (iv) Wider decision boundaries. Bayesian classifiers estimate class probabilities based on observed feature distributions. When both the BI and YI are used, the classifier can establish more refined decision boundaries, reducing misclassification between different browning stages, thereby increasing the classification accuracy. (v) Dependence on the context. Pear browning is affected by a number of physiological factors, including the storage time, temperature, and oxygen levels. The Bayesian framework integrates prior knowledge and feature dependencies (i.e., a high BI may have a different meaning if the YI is also high, as this may indicate senescence rather than enzymatic browning). In conclusion, using the BI and YI together provides a more comprehensive and informative input to the Bayesian classifier. This leads to improved classification accuracy by capturing interdependencies between the two indices and improving the model’s ability to distinguish between different types of browning.

**Figure 9:**
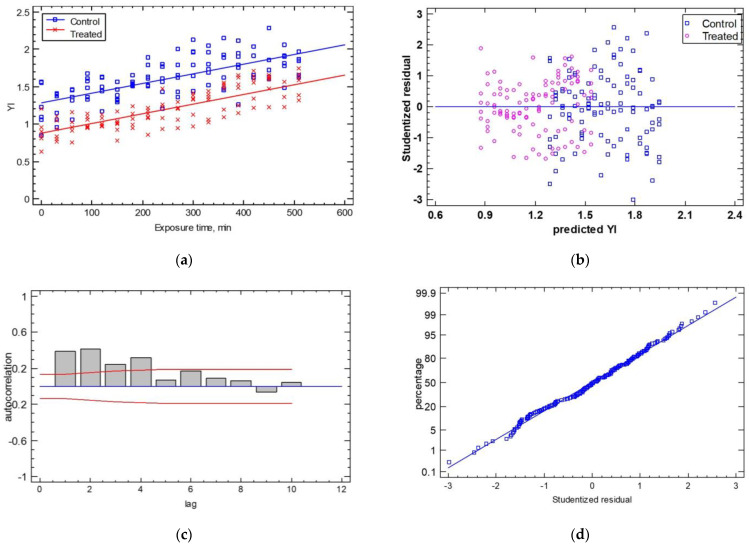
(**a**) The temporal evolution of the YI shape factor for the control and treated samples. The experimental data are described as two lines, which have the same slope and different intercepts (YI, t = 0). Residual test plots are presented for (**b**) homoscedasticity, (**c**) autocorrelation, and (**d**) normality.

The comparison of the regression lines regarding the shape factor of the YI with the exposure time, and using the two cases (control vs. treated samples) produced a good prediction (R^2^_adj_ = 70.33% and SEE = 0.18) (Εquation (8)). The ANOVA of the line intercepts and slopes demonstrated that only the line intercepts exhibited a significant difference (*p* ≤ 0.05), while the line slopes were not significantly different (*p* = 0.2310 > 0.05) (see Figure 9a). The statistical analysis of the control and the treated samples found average shape factors for the YI of 1.62 ± 0.3 and 1.20 ± 0.2, respectively, with a coefficient of variation of 18.69% and 21.56% for the control and treated samples, respectively.

The analysis of the statistical hypotheses concerning the calculated residuals produced good results in terms of homoscedasticity, autocorrelation, and normality, as shown in Figure 9b–d. For homoscedasticity, the residuals were relatively close, with no systematic pattern. The most significant feature of this plot was that the “treated” samples were more closely distributed around the horizontal line than the “control” samples. For the autocorrelation hypothesis, few bars extended beyond the probability limits, indicating a significant dependence between residuals separated by the specified “lag”. Finally, the normality test showed that the deviations followed a normal distribution as they fell along a straight line. The estimates of the coefficients of the linear Equation (8) were (estimate ± standard error) a = 1.280 ± 0.02 and b = 0.00125 ± 0.00008 and for Equation (9), a = 0.885 ± 0.02 and b = 0.00125 ± 0.00008 (*p*-value ≤ 0.05).

Control: YI = 1.280 + 0.00125 × Exposure Time (min)(8)

Treated: YI = 0.885 + 0.00125 × Exposure Time (min)(9)

**Figure 10:**
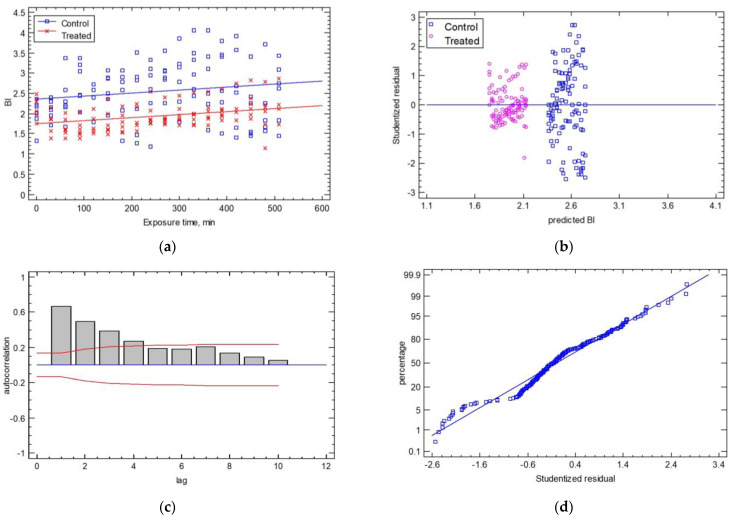
(**a**) The temporal evolution of the BI shape factor for the control and treated samples. The experimental data are described as two lines, which have the same slope and different intercepts (BI, t = 0). Residual test plots are presented for (**b**) homoscedasticity, (**c**) autocorrelation, and (**d**) normality.

The comparison of the regression lines for the shape factor of the BI with the exposure time and the cases of control vs. treated samples produced a poor prediction (R^2^_adj_ = 26.62% and SEE = 0.54). The ANOVA of the intercepts and slopes showed that only the intercepts of the two regressed lines were significantly different (*p* ≤ 0.001), while the line slopes had a *p*-value of 0.7616 (see Figure 9a). The statistical analysis of the control samples vs. the treated samples found that the average BI was 2.55 ± 0.7 for the control samples and 1.94 ± 0.3 for the treated samples with a coefficient of variation of 27.58% for the control samples and 17.17% for the treated samples. The analysis of the statistical hypotheses concerning the calculated residuals gave adequate results in terms of homoscedasticity, autocorrelation, and normality, as shown in Figure 10a. For homoscedasticity, the residuals were barely over the range of the residuals and showed a non-systematic pattern. In Figure 10b, the treated samples were significantly closer to the horizontal line than the control samples. For the autocorrelation hypothesis, few bars extend beyond the probability limits, indicating a significant dependence between residuals separated by the specified “lag” (Figure 10c). Finally, the normality test showed that the deviations followed a normal distribution as they fell along a straight line (Figure 10d). The estimates of the coefficients of the linear Equation (10) were (estimate ± standard error) a = 2.380 ± 0.08 and b = 0.00076 ± 0.0002 and for Equation (11), they were a = 1.721 ± 0.07 and b = 0.00076 ± 0.0002 (*p*-value ≤ 0.05).

Control: BI = 2.380 + 0.00076 × Exposure Time(10)

Treated: BI = 1.721 + 0.00076 × Exposure Time(11)

The control data for the shape factors of the BI demonstrated the highest coefficient of variation (>10%), contributing significantly to the lack of accuracy observed in Equation (10). This response was not observed with the shape factors of the YI.

As demonstrated by the slope values of Equations (1) and (2), the YI increased at a rate twice (0.00125) that of the BI (0.00076). This showed that the yellowing process took place at a significantly faster rate than the browning process, although the more complex the biological processes are, the more difficult it is to draw conclusions. The problems and limitations that may have caused the low estimation accuracy are discussed below. 

The BI depends on combinations of L*, a*, and b* values in the CIE Lab* colour space; the index was developed to assess red-brown hues. However, its implementation may experience limitations in representing small or uneven discolouration. Pear browning is frequently influenced by multiple pigments, including quinones (reddish-brown), chlorophyll (green), and carotenoids (yellow-orange). These pigments may not align with the assumptions underlying the BI, potentially leading to misrepresentations. It is important to note that the BI assumes uniform browning across the sample; however, this may not always be the case in practice. Non-uniform browning can lead to inaccuracies when the sample’s overall colour does not match the averaged CIE Lab* values. Enzymatic browning often starts in areas like wounds or bruises resulting in non-homogeneous changes. The BI is not always able to evaluate the relative contributions of red and yellow to the final browning process. This can lead to over- or under-estimation of browning. This limitation is based on the fact that enzymatic browning primarily produces red-brown hues; however, concurrent yellowing from carotenoid oxidation or chlorophyll degradation can interfere with the accuracy of the browning estimation.

The YI is focused on the b* (yellow–blue) axis, which might not show the full range of browning since it involves dark pigments. Browning is usually associated with reddish-brown pigments, which are the result of the enzymatic oxidation of phenolic compounds. However, it is important to note that the YI’s design does not always accurately capture these pigments. The YI does not account for L* (lightness), which is critical for distinguishing between light and dark browning stages. It is important to note that browning in pears frequently results in a darker colouration, and the failure to account for lightness changes can lead to an underestimation of the browning intensity. The YI can be affected by the fruit’s natural yellow pigments, such as carotenoids, or by the fruit’s initial colour (green or yellow). Pears with more yellow to begin with might show artificially high YI values even before they start to brown. The YI has a limited response to changes in red-brown pigments or variations in the yellow spectrum. As the enzymatic browning progresses, the red and brown hues become predominant, and the YI cannot fully capture this due to its narrow focus on yellowness.

It is important to note that browning and yellowing indices can be affected by the lighting conditions, observer’s angle, and glossiness of the pear surface. It has been shown that pears exhibit structural colour variations and inconsistent measurement setups. All these factors can distort the CIE Lab* or XYZ values employed for BI and YI computation. The calculation of both the BI and YI relies on averaged colour values, which mask local variations in browning intensity across the fruit surface. Enzymatic browning frequently initiates in localized regions, such as bruises, resulting in uneven discolouration. The proposed image analysis methodology, based on the BI and YI, provides two types of information. Firstly, it provides qualitative information regarding the quality of the pear samples based on the surface colour. Secondly, it provides quantitative information regarding the rate at which this visual quality of the samples is changing due to oxidation or enzymatic browning. The analysis underlined the limitations of employing a single colour assessment index, such as the BI or the YI, to evaluate a multi-faceted phenomenon such as fresh-cut pear browning, particularly in conjunction with other critical indices.

## 5. Discussion

The image analysis used in this study was based on a digital 2-dimensional camera (Konica Minolta DiMAGE Z6; Konica Minolta, Inc., Tokyo, Japan), along with a light booth (Graphic Lite GLE PDV-3eTR, Graphic Technology, Inc., New York, NY, USA) equipped with a D65 lighting system (L317, Graphic Technology, Inc., New York, NY, USA). This ensured the acquisition of high-quality digital images in a constant lighting environment. This experimental setup is essential if high-level information needs to be extracted from an object or regions of interest since a range of consecutive image processes needs to be carried out. These processes included image thresholding, segmentation, transformation of the RGB model to the CIE Lab* model, and transformation of L*, a* and b* values to BI and YI values. Finally, the statistical analysis was based on tri-parameter Weibull modelling, and statistical inference was conducted in the last processing step. The overall process demands quite considerable computation power and expertise in mathematical modelling and statistical inference. To obtain high-quality data from the image processing, expensive equipment is required, i.e., the cost of a digital camera can exceed EUR 1000, the professional lighting setup can exceed EUR 2000, and, a programming and numeric computing platform is necessary since colour model transformations will be carried out along with the statistical analyses. The latter steps require engineering expertise given the involvement of complex algorithms. The stochastic modelling employed in this particular section of the study demonstrated a high level of proficiency in conducting precise and controlled assessments, thereby providing quantitative indices such as the Browning Index and the Yellowing Index. These indices were shown to be effective tools for the monitoring of enzymatic changes over time. Moreover, this approach was able to correlate image data with physical and chemical changes in pears, thereby supporting detailed quality assessments. The fundamental advantage of processing digital images in a stochastic manner is that it does not require extensive training datasets, which simplifies its implementation. However, image processing techniques have weaknesses as well. For example, they are influenced by the sample preparation and the imaging conditions, which can affect reproducibility. They also exhibit difficulties in terms of adaptability to more complex tasks or when datasets with overlapping characteristics are used.

The versatility of our model was demonstrated by its implementation on both a Raspberry Pi device equipped with a basic web camera and a high-end smartphone (an iPhone 13, Apple Inc., Cupertino, CA, USA) [30]. A more in-depth analysis and more precise predictions were facilitated by the superior camera capabilities of the iPhone 13 smartphone compared to those obtained with the Raspberry Pi setup. This finding highlights the significant role that hardware quality plays in model performance. Nevertheless, it should be noted that this configuration is neither the most optimal nor viable option, as smartphones are intended for a completely different use, consume more energy, and in this specific configuration, are always reliant on an internet connection and the cloud.

Despite the data augmentation and normalization steps performed at the pre-processing stage, the results revealed that the camera’s proximity to the sample still influenced the accuracy of the classifications. The ability of the model to deliver decisions within a few milliseconds is an undisputable advantage for real-time applications. However, to improve its performance, more than one classification decision can be considered for a specific sample (e.g., from more than one camera placed at different angles) before making a final decision. In any case, for effective deployment of ML models in real-world applications, the results of this study highlight the importance of deploying well-designed and trained decision-making processes and tailoring them for the appropriate hardware.

The CPU utilization of the Raspberry Pi single-board computer increased by approximately 10% due to the NN model execution and the prior execution of image streaming operations. The time required for a decision-making process to occur ranged from 10 ms to 13 ms on the Raspberry Pi unit. The financial cost of the prototype automatic classification system, based on machine learning methods, was approximately EUR 100. This cost analysis includes the Raspberry Pi 4 (Raspberry Pi Foundation, Cambridge, UK) (EUR 60), the power supply (EUR 10), the Logitech HD C270 camera (Logitech International S.A., Lausanne, Switzerland) [28] (EUR 20), and the micro-SD card (EUR 10). The utilization of a camera and a better analysis method may slightly increase the cost by EUR 10 to 20. The cost of cloud-based solutions utilizing either the Raspberry Pi or a smartphone can start at EUR 100, with the price increasing with the specifications of the smartphone. Nevertheless, the cloud solution is not the optimal choice due to its inherent privacy concerns, dependencies on network connectivity, and dependency on external platforms. The pilot implementation we presented highlights the feasibility of achieving satisfactory results utilizing generally applicable methods and low-cost equipment compared with typical industrial cameras (RGB + NIR) that cost at least 50 times more [44]. Furthermore, as indicated by our previous research [45], the Raspberry Pi unit can be combined with neural network processing acceleration modules (e.g., the Intel Movidius NCS2 module) to drastically improve its performance whenever necessary. On the other hand, defining the exact requirements for fluent performance is also dependent on the electromechanical characteristics of the overall food production system that may vary drastically but is beyond the scope of this work.

Based on the strengths and weaknesses of machine learning and stochastic-based methods that we discussed, we think that there is fertile ground for future investigations combining both approaches. A hybrid system integrating both methods could improve automated quality control in food processing. Towards this goal, key parts of the stochastic modelling algorithms could be modified in order to be executed by the embedded hardware that is mainly responsible for the machine learning part. The new system could first use a CNN-based model to quickly classify fruit slices as acceptable or brown, enabling real-time sorting in automated processing lines. Selected samples will then undergo further analysis using stochastic modelling with BI and YI calculations to ensure accurate tracking of enzymatic changes and refined results. The system can also continuously utilize feedback from the stochastic model to improve the quality of the input data used for CNN model training, thus achieving adaptability to different fruit varieties and conditions. Consequently, by complementing the automated machine learning parts with algorithms implementing the stochastic modelling formulas, a hybrid approach could further enhance the robustness and applicability of image analysis systems in agricultural applications, thus increasing the efficiency and consistency in food processing and ensuring reliable quality control.

## 6. Conclusions

In this study, two image analysis methodologies for the classification of fresh and enzymatically browned pear slices were evaluated. The first methodology employed convolutional neural networks (CNNs) while the second methodology utilized stochastic modelling techniques. Both approaches for agricultural image analysis exhibited advantages and disadvantages. The CNN-based methodology achieved a high accuracy and had the capacity to perform real-time decision-making, which makes it ideal for tasks in automated processing lines. Despite the limited dataset, the application of transfer learning and data augmentation during the training resulted in a satisfactory accuracy of 96.6% for the ML model. Another advantage of the CNN-based methodology is that it can be adapted to other classification problems by simply adjusting the training settings and using a more appropriate dataset. Furthermore, the selection of cost-effective components, such as a standard USB camera and a Raspberry Pi, enables the fast and practical incorporation of this mechanism into actual production line systems. Although this method has advantages, there is a potential risk of overfitting the model when there training data are limited and the reliability of system may also be affected by environmental variability and inconsistent imaging conditions. However, the environment the model will work in (food processing lines) is characterized by controllable/constant light conditions, thus eliminating the risk of potential decision failures. On the other hand, stochastic modelling provides quantitative indices (i.e., the Browning Index and Yellowing Index), which can assist in effectively monitoring enzymatic changes over time, and do not require extensive training datasets. Limitations still exist in terms of scalability to more complex tasks or datasets with overlapping characteristics, and it is vulnerable to the variability and imaging conditions of the dataset. Additionally, in contrast to the CNN-based method, stochastic modelling requires exhaustive manual tuning of the parameters and requires the participation of persons with expertise in mathematical modelling. Employing the BI and YI together proved to be an informative input to the Bayesian classifier, improving the classification accuracy. The combination of these two indices allowed the model to capture interdependencies between them and improved the its ability to distinguish between different types of browning.

In summary, CNN-based methodologies are suitable for high-throughput real-time classification tasks for a wide variety of fruit products, while stochastic modelling is better suited for laboratory-oriented precise evaluations of specific optical or chemical phenomena. In conclusion, it is important to highlight that, while computer vision systems can exhibit automation potential and suitability for non-destructive evaluation, their performance is still dependent on the expertise of food scientists and signal-processing specialists. Thus, the integration of multidisciplinary expertise remains a critical factor for the development of more adaptable and reliable automated agricultural imaging applications.

## Figures and Tables

**Table 1:** Confusion matrix for binary classification purposes.

	Predicted Positive Class	Predicted Negative Class
**Actual Positive Class**	TP	FN
**Actual Negative Class**	FP	TN

**Table 2:** The confusion matrix for the testing of the deployed model.

	Predicted Label: Brown	Predicted Label: Good
**Actual label: brown**	2080	137
**Actual label: good**	0	1863

**Table 3:** The performance metrics for each class of the deployed model.

	Precision	Recall (or Sensitivity)	F1-Score
**Brown**	100%	93.82%	0.968
**Good**	93.15%	100%	0.965

## Data Availability

The data presented in this study are available upon request.
